# Characterization and comparison of the fecal bacterial microbiota in Red Back Pine Root Snake (*Oligodon formosanus*) and Chinese Slug-Eating Snake (*Pareas chinensis*)

**DOI:** 10.3389/fmicb.2025.1575405

**Published:** 2025-04-16

**Authors:** Xiao Cong, Xiangnan Liu, Dan Zhou, Yunfeng Xu, Jinru Liu, Fei Tong

**Affiliations:** ^1^Guangdong Provincial Key Laboratory of Organ Donation and Transplant Immunology, The First Affiliated Hospital, Sun Yat-sen University, Guangzhou, China; ^2^Department of Infectious Diseases and Public Health, Jockey Club College of Veterinary Medicine and Life Sciences, City University of Hong Kong, Kowloon Tong, Hong Kong SAR, China; ^3^Fu Shun Vocational Technology Institute, Fushun, China

**Keywords:** Red Back Pine Root Snake (*Oligodon formosanus*), Chinese Slug-Eating Snake (*Pareas chinensis*), gut microbiota, microbial function, bacterial pathogens

## Abstract

**Introduction:**

The gastrointestinal tracts and oral cavities of animals harbor complex microbial communities that assist hosts in nutrient absorption and immune responses, thereby influencing behavior, development, reproduction, and overall health.

**Methods:**

We utilized metagenomic sequencing technology to conduct a detailed analysis of the fecal bacterial communities of six Red Back Pine Root Snakes (*Oligodon formosanus*, XT) and three Chinese Slug-Eating Snakes (*Pareas chinensis*, Z) individuals. The microbial composition was assessed through taxonomic profiling, alpha diversity analysis, and functional annotation using the Kyoto Encyclopedia of Genes and Genomes (KEGG) database.

**Results:**

The results indicated that Proteobacteria, Bacteroidetes, Firmicutes, Verrucomicrobia, Actinobacteria, and Fusobacteria were the dominant phyla in XT samples, while Z samples additionally contained Patescibacteria. Alpha diversity analysis revealed significant differences in species abundance at the family level, with Z samples exhibiting higher microbial richness than XT. Furthermore, KEGG analysis showed that XT had higher functional gene abundance in pathways related to transcription, translation, environmental adaptation, membrane transport, cellular communities (prokaryotes), motility, and replication/repair compared to Z.

**Discussion:**

This study provides a comparative analysis of their gut microbiomes, offering valuable insights for future research on zoonotic diseases, host-microbe interactions, and ecological, evolutionary, behavioral, and seasonal influences on snake microbiota. These findings contribute to a broader understanding of microbial ecology in reptiles and its implications for conservation and disease dynamics.

## 1 Introduction

In China, more than 205 species of snakes inhabit various environments, including grasslands, wetlands, forests, farmlands, sand plateaus, and marine areas (Zhou and Jiang, 2004). These snakes have a diverse diet and feed on mammals, birds, reptiles, amphibians, insects, small invertebrates, and fish. Their living environment, behavioral traits, physiological characteristics, and dietary habits all influence the microbial communities found in their oral cavity and intestines, leading to a rich microbiota in these areas (Costello et al., 2010; Tang et al., 2019). In most animals, the gastrointestinal (GI) tract contains billions of microorganisms, which help the host to absorb nutrients and produce immune responses. These immune responses, in turn, influence the host’s behavior, development, reproduction, and other characteristics (Colston and Jackson, 2016; Ellegaard and Engel, 2016; Ezenwa et al., 2012). However, current research on the intestinal microbiota of reptiles is limited to a few species, including *Naja naja*, *Ophiophagus hannah*, *Python molurus* (Krishnankutty et al., 2018), *Rhabdophis subminiatus* (Tang et al., 2019), *Python bivittatus* (Costello et al., 2010), *Naja atra*, *Ptyas mucosa*, *Elaphe carinata*, *Deinagkistrodon acutus* (Zhang et al., 2019), and *Ptyas mucosa* (Qin et al., 2019). Therefore, exploring the bacterial diversity in the gut is of great significance for studying their physiology and genetics (Hu et al., 2024).

The Red Back Pine Root Snake (*Oligodon formosanus*) is a nonvenomous species native to Taiwan in China. It belongs to the family Colubridae and is harmless, non-venomous snakes (Huang et al., 2011). The Red Back Pine Root Snake is characterized by its distinctive reddish or orange stripe running along its back, which is where it gets the name “Red-back.” Its coloration helps it to blend in with the forest floor. This species feeds on small vertebrates like eggs of lizards, frogs, and possibly other small animals found in its habitat. It is a terrestrial species, meaning it spends most of its time on the ground. It is nocturnal or crepuscular, and is most active during the night or twilight hours. The Red Back Pine Root Snake is nonvenomous and relies on constriction to subdue its prey. This species is often considered beneficial for controlling small vertebrate populations.

The Chinese Slug-Eating Snake (*Pareas chinensis*) is a nonvenomous species native to China, as well as to parts of Vietnam and Laos. It belongs to the family Pareidae, a group of snakes known for their specialized feeding habits (Gong et al., 2023). The Chinese Slug-Eating Snake is a small, slender species that is typically found in forests, particularly in lowland and mountainous regions (Guo et al., 2020). It thrives in moist environments and is commonly associated with vegetation and leaf litter, where its cryptic coloration—usually brown, gray, or olive with darker markings—helps it to blend into its surroundings. As its name suggests, this snake primarily feeds on slugs, a relatively unusual diet for snakes. It has developed specialized feeding adaptations, including constriction, to subdue its prey. This unique diet makes the Chinese Slug-Eating Snake an interesting subject for studying diet specialization. The species is terrestrial and nocturnal, most active at night and often hiding under leaf litter or within vegetation during the day. Ecologically, the Chinese Slug-Eating Snake plays a crucial role in controlling slug populations, contributing to the balance of its habitat’s ecosystem.

The dynamic interactions between hosts and their microbiomes profoundly influence metabolic regulation and environmental adaptation (Wang et al., 2022a; Zhu et al., 2021; Zhu W. et al., 2022). In addition to host genetic variation and dietary patterns, factors such as geographical distribution, habitat environment, and intestinal physicochemical characteristics (e.g., pH, oxygen levels) shape the composition of gut microbiota (Trevelline et al., 2019). This long-term co-evolutionary equilibrium enables microbial communities to act as critical mediators in host adaptation by regulating metabolic activity, nutrient absorption, immune responses, and behavioral ecology (Wang et al., 2022b; Zhu J. et al., 2022). Studies indicate that increased gut microbial diversity is positively correlated with enhanced metabolic efficiency and energy acquisition, whereas reduced diversity or overgrowth of harmful taxa may trigger metabolic disorders and inflammatory responses (Cotillard et al., 2013; Ley et al., 2008).

Among reptiles, the snake intestinal system serves as an ideal model for studying extreme environmental adaptation due to its unique physiological traits. For instance, pythons undergo dramatic intestinal regeneration after feeding, and the activated signaling pathways (e.g., Wnt/β-catenin) share striking similarities with metabolic reprogramming pathways observed in humans following Roux-en-Y gastric bypass surgery. This discovery not only reveals evolutionarily conserved mechanisms of intestinal regeneration but also identifies potential therapeutic targets for metabolic diseases (e.g., type 2 diabetes) and intestinal dysfunctions (e.g., inflammatory bowel disease). Notably, a specialized intestinal cell type called BEST4^+^—present in both pythons and humans but absent in model organisms like mice—plays a central regulatory role in early-stage regeneration by facilitating lipid transport and metabolism. The snake model thus offers unique insights into the evolutionary function of these cells in vertebrates (Westfall et al., 2024). In summary, research on snake intestines not only advances our understanding of molecular mechanisms underlying extreme physiological adaptation but also establishes an interdisciplinary platform bridging fundamental medicine, ecological conservation, and technological innovation, potentially offering transformative solutions for human disease treatment and animal husbandry optimization.

In this study, we utilized metagenomic analysis to investigate the diversity of gut communities in the Red Back Pine Root Snake and the Chinese Slug-Eating Snakes, with the aim to explore the composition of microbial communities and their potential functions to enhance our understanding of ecology, host interactions, and adaptive evolution.

## 2 Materials and methods

### 2.1 Sample site collection

In June 2021 in Guangdong province, we captured six Red Back Pine Root Snake (XT species) and three Chinese Slug-Eating Snakes (Z species) in the forests and hills of Zhanjiang City, Guangdong Province, China. To prevent sample contamination, we placed the two types of snakes in separate buckets disinfected with 75% alcohol for immobilization and collected feces samples from their intestines using rectal swabs (Tian et al., 2022). At the same time, all swabs were placed in RNase-free centrifuge tubes, and after nucleic acid extraction, they were immediately transported on dry ice to Sangon Biotech (Shanghai) Co., Ltd. Finally, the captured snakes were released back into the wild.

### 2.2 DNA extraction and database sequencing

According to the manufacturer’s instructions, a Stool DNA Kit (OMEGA, United States) was used to separately extract pathogen/total microbial DNA from fecal samples (Sun et al., 2013). Metagenomic shotgun sequencing libraries were constructed at Sangon Biotech (Shanghai). In summary, after extracting the total DNA from the samples, specific primers with barcodes were synthesized based on the full-length primer sequences. PCR amplification was conducted, followed by purification, quantification, and normalization to create a sequencing library (SMRT Bell). The constructed library was subjected to quality control, and those that passed were sequenced using PacBio Sequel. The data output from PacBio Sequel was in BAM format, and CCS files were exported using SMRT Link analysis software. Data from different samples were identified based on barcode sequences and converted into FASTQ format.

### 2.3 Data preprocessing

After exporting the PacBio output data as CCS files (CCS sequences obtained using the SMRT Link tool provided by PacBio), three main steps were performed. (1) CCS identification: The lima v1.7.0 software was used to identify CCS sequences through barcodes, resulting in Raw-CCS sequence data. (2) CCS filtering: The cutadapt 1.9.1 software was utilized to recognize and remove primer sequences, as well as to perform length filtering, yielding Clean-CCS sequences that do not contain primer sequences. (3) Chimeric sequence removal: The UCHIME v4.2 software was used to identify and remove chimeric sequences, resulting in Effective-CCS sequences. Information analysis included the classification of features (OTUs, ASVs), species annotation and taxonomic analysis, diversity analysis, differential analysis, correlation analysis, and functional prediction analysis.

### 2.4 Species annotation and taxonomic analysis

Using the genome sequences of all bacteria, archaea, fungi, viruses, protists, and algae from the NCBI database (ftp://ftp.ncbi.nlm.nih.gov/refseq/TargetedLoci/), the clean reads from each sample were classified taxonomically. Classification was performed at seven phylogenetic levels: domain, phylum, class, order, family, genus, species, and unclassified. Bracken^2^ was used to calculate the abundance of each taxon. The relative abundance at a specific taxonomic level represents the cumulative abundance of all species classified at that level.

### 2.5 Diversity analysis

Diversity analysis includes both alpha and beta diversity analyses. Alpha diversity refers to the diversity within a specific area or ecosystem. Common metrics for measuring microbial richness include the Chao 1 and Ace richness estimators. The metrics for measuring microbial diversity include the Shannon–Wiener diversity index, Simpson diversity index, and phylogenetic diversity index. QIIME2 is the most commonly used software for Alpha diversity. Beta diversity analysis is used to determine changes in species composition over time and spatial scales. Sample principal coordinate analysis (PCoA) and the PERMANOVA/Anosim graph were plotted based on the R language platform to analyze the correlation between environmental factors and sample composition. PCoA analysis was performed under ordination analysis. The ordination process rearranges these samples in a visual low-dimensional space or plane, such that the distances between samples most accurately reflect their relationships within the scatter plot.

### 2.6 Correlation and association analysis

Based on the abundance and variation of each species in the samples, Spearman rank correlation analysis (default method) was conducted, and a correlation network was constructed by selecting data with a correlation > 0.1 and a *p*-value < 0.05.

### 2.7 Functional prediction analysis

PICRUSt2 is a computational method that uses marker gene data and a reference genome database to predict the functional composition of environmental microbes. The functional potential of microbial communities during phylogenetic processes is predicted based on the correlation between phylogeny and function using IMG microbial genome data.

## 3 Results

### 3.1 General characteristics of sequence data

In this study, we performed metagenomic sequencing of fecal samples from six Red Back Pine Root Snakes (*Oligodon formosanus*, XT) and three Chinese Slug-Eating Snakes (*Pareas chinensis*, Z) using the Illumina NovaSeq 6000 platform. A total of 61,449 raw CCS reads were obtained, with an average sequence length per fragment ranging from 1,439 to 1,460 bp after quality control filtering. After primer identification and removal, the number of clean CCS reads was 54,980. After length filtering and the removal of chimeric sequences, 53,687 effective CCS reads were retained for subsequent analyses.

**Figure 1:**
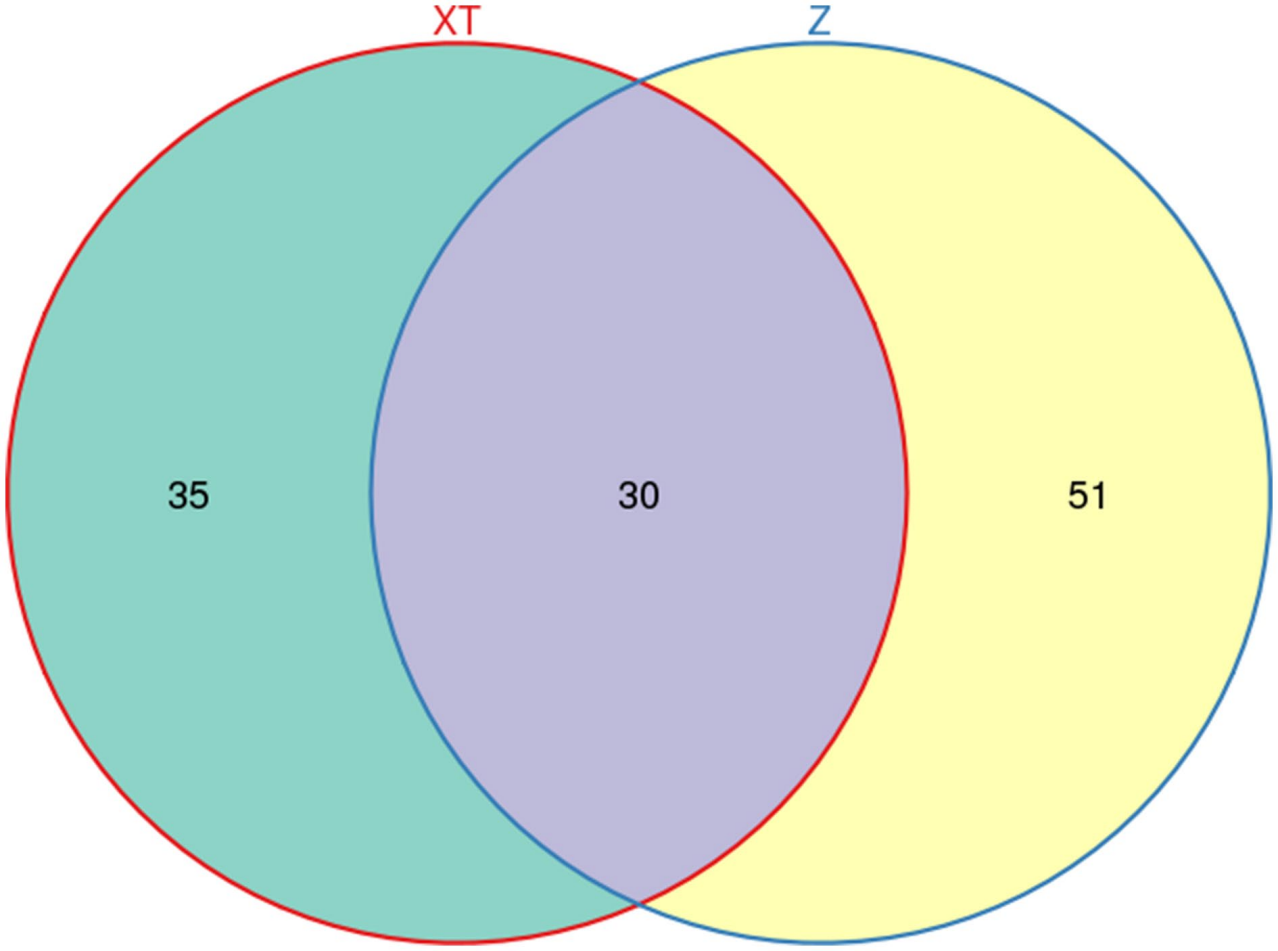
Diagram showing the number of shared features between the two snake species and the distribution of unique features for each species.

A Venn diagram illustrates the number of shared and unique features between samples, providing a visual representation of their overlap. Based on Venn diagram analysis, 30 common features were found to be shared between the Red Back Pine Root Snakes and the Chinese Slug-Eating Snakes, whereas 35 and 51 distinctive features were unique to the two species, respectively (Figure 1).

### 3.2 Gut microbial diversity and community composition

**Figure 2:**
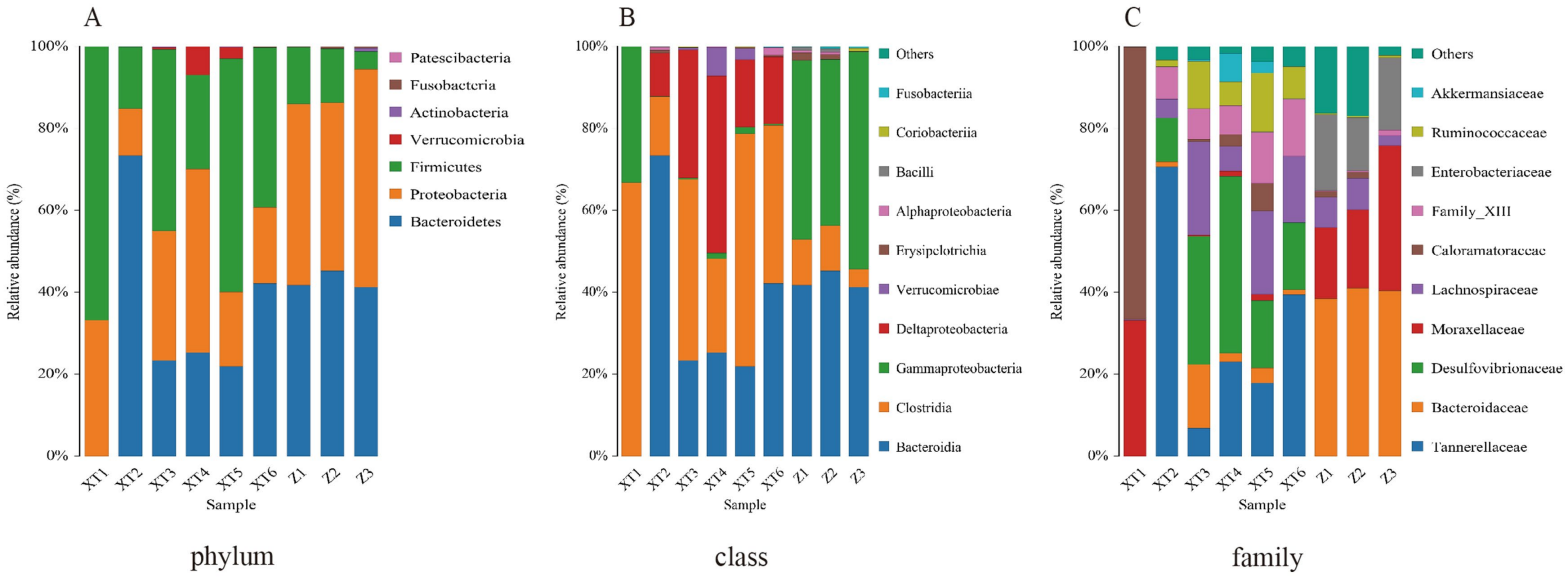
Relative abundance ratios of microbial communities at the phylum **(A)**, class **(B)**, and family **(C)** levels in fecal samples of the Red Back Pine Root Snake (*Oligodon formosanus*, XT) and the Chinese Slug-Eating Snake (*Pareas chinensis*, Z).

As shown in Figure 2A, at the phylum level, Firmicutes, Proteobacteria, and Bacteroidetes were identified as the dominant phyla across all nine snake species, consistent with findings from most snake gut microbiota studies. At the class level, Clostridia was determined to be the dominant class in all samples, though its relative abundance was notably higher in the XT samples compared to the Z samples. Bacteroidia emerged as the dominant class in all samples except XT1, while Gammaproteobacteria dominated in XT1 and Z1–3, which may be attributed to individual variation. Deltaproteobacteria was the dominant class in XT2–6, and this substantial divergence likely reflects species-specific differences (Figure 2B). Similarly, at the family level (Figure 2C), Tannerellaceae, Desulfovibrionaceae, Family_XIII, Lachnospiraceae, Ruminococcaceae, and Bacteroidaceae were identified as dominant families in XT2–6, whereas only Caloramatoraceae and Moraxellaceae were observed in XT1, potentially due to inter-individual variability. In contrast, the primary families in Z1–3 included Bacteroidaceae, Moraxellaceae, Enterobacteriaceae, and Lachnospiraceae, which differed significantly from those in XT samples, further supporting species-related divergence (the specific data information can be found in Supplementary Document 1).

These results indicate that the gut microbiota of the two snake species exhibit differences, which may be closely related to their distinct living environments. Differences in habitat likely lead to variations in diets, ultimately resulting in differences in gut microbiota.

### 3.3 Comparison of bacterial community structure at the phylum and family levels

**Figure 3:**
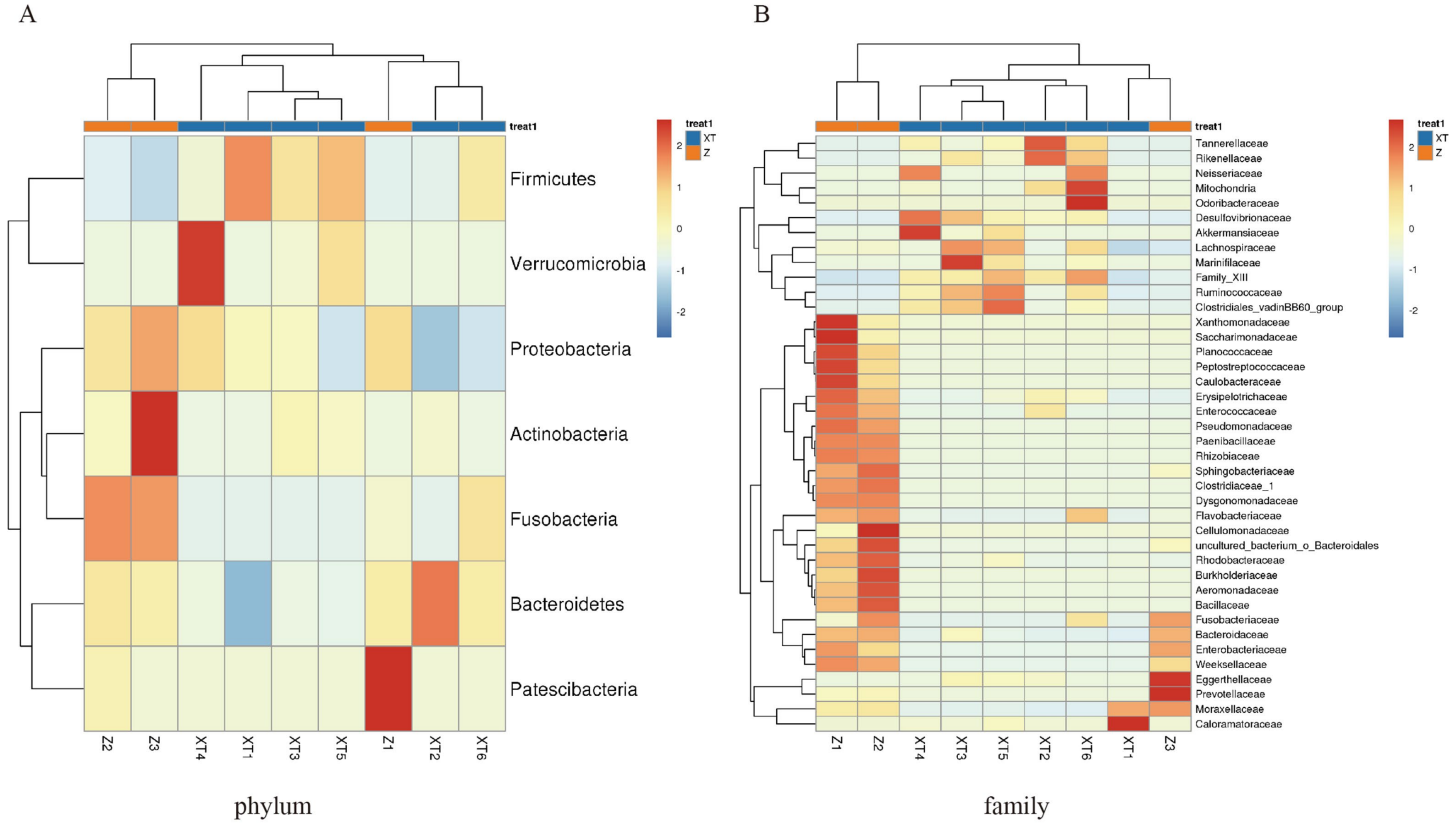
Heatmap of the overlapping abundance readings for each sample. **(A)** Abundance at the phylum level for each sample. **(B)** Abundance at the family level for each sample.

The heatmap analysis revealed that the structure of different bacterial communities is influenced by the snake species. At the phylum level, the relative abundance of Firmicutes was significantly higher in XT samples than in Z samples; Verrucomicrobia showed a higher relative abundance only in XT4–5, whereas Patescibacteria was more abundant in Z1 and showed no significant differences in other samples. Fusobacteria and Proteobacteria had a significantly higher relative abundance in the Z samples compared to XT samples; Actinobacteria was more abundant in the Z3 samples, but showed no significant differences between the other sample groups. Bacteroidetes was relatively abundant only in XT2, whereas its relative abundance was lower in the other XT sample groups (Figure 3A).

At the family level, our analysis revealed differences between the Z and XT samples. The Z samples exhibited high abundances of the families Xanthomonadaceae, Saccharimonadaceae, Planococcaceae, Peptostreptococcaceae, Caulobacteraceae, Erysipelotrichaceae, Enterococcaceae, Pseudomonadaceae, Paenibacilaceae, Rhizobiaceae, Sphingobacteriaceae, Clostridiaceae_1, Dysgonomonadaceae, Flavobacteriaceae, Cellulomonadaceae, uncultured_bacterium_O_Bacteroidales, Rhodobacteraceae, Burkholderiaceae, Aeromonadaceae, Bacillaceae, Fusobacteriaceae, Bacteroidaceae, Enterobacteriaceae, and Weeksellaceae, all of which showed notably lower abundances in the X samples. In contrast, the X samples were dominated by families such as Desulfovibrionaceae, Akkermansiaceae, Lachnospiraceae, Marinifilaceae, Family XIII, Ruminococcaceae, Clostridiales_vadinBB60_group, Tannerellaceae, Rikenellaceae, Neisseriaceae, Mitochondria, and Odoribacteraceae. These distinct microbial community profiles likely reflect adaptations to their divergent living environments (Figure 3B).

### 3.4 Alpha diversity analysis

**Figure 4:**
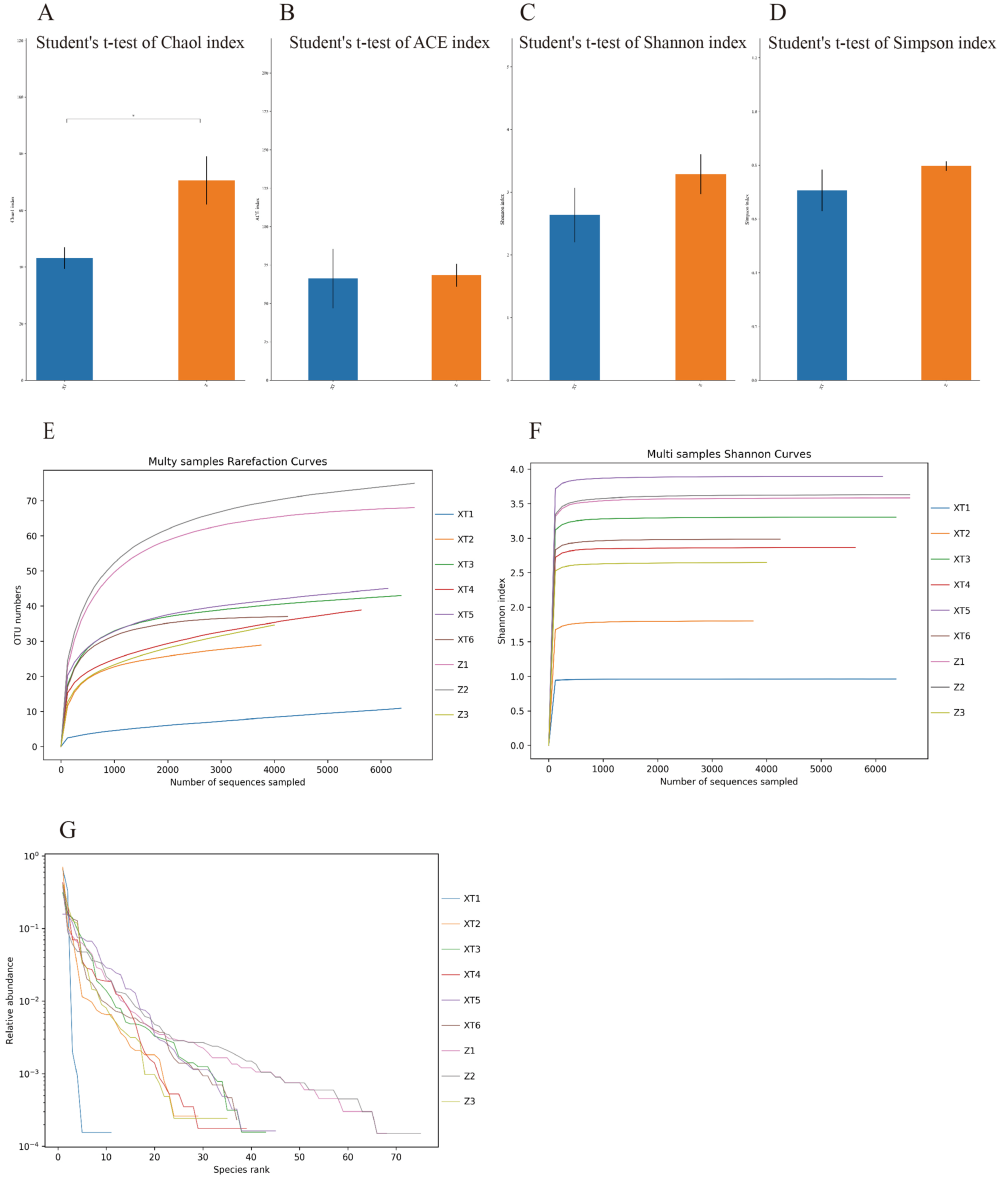
Alpha diversity index statistical chart. **(A)** Chao 1 richness estimator; **(B)** Ace richness estimator; **(C)** Shannon-Wiener diversity index; and **(D)** Simpson diversity index. **(E)** Rarefaction curve; **(F)** Shannon index curve; and **(G)** Rank abundance curve. *p < 0.05; **p < 0.01; ***p < 0.001.

Using QIIME2 software to analyze the samples’ alpha diversity, we found a significant difference in the Chao 1 richness estimator (Figure 4A), indicating a clear difference in diversity between the two microbial communities. The Z sample exhibited higher alpha diversity, suggesting a richer microbial diversity. We also analyzed the Ace richness estimator, Shannon-Wiener diversity index, and Simpson diversity index for the two samples and found no significant differences in these three metrics, but the values for the Z sample were slightly higher than those for the XT sample (Figures 4B–D), indicating that overall, the microbial diversity of the Z sample was higher than that of the XT sample. Specific data information can be found in Supplementary Document 2.

The rarefaction, Shannon index, and rank abundance curves can all be used to analyze the species diversity in the samples. The rarefaction curve serves as a measure of whether the sequencing depth of each sample is sufficient. From Figure 4E, we can see that all curves approach a plateau, indicating that they accurately reflect the microbial community and that the results are sufficient to estimate microbial diversity. The Shannon index, similar to the rarefaction curve, also reflects microbial diversity. The curves were plotted using Mothur software. A larger Shannon index indicates a greater number of species and higher richness, suggesting that the samples encompass the vast majority of microbial species information (Figure 4F). Additionally, the rank abundance curve displays the abundance of features from each sample, which is sorted by size and plotted based on their relative abundance. A wider curve indicates a richer species composition, whereas a flatter curve indicates a more even species composition (Figure 4G). The analysis results show that all nine snake species exhibit high species richness; however, overall, the Z samples exhibit higher species richness.

### 3.5 Beta diversity analysis

**Figure 5:**
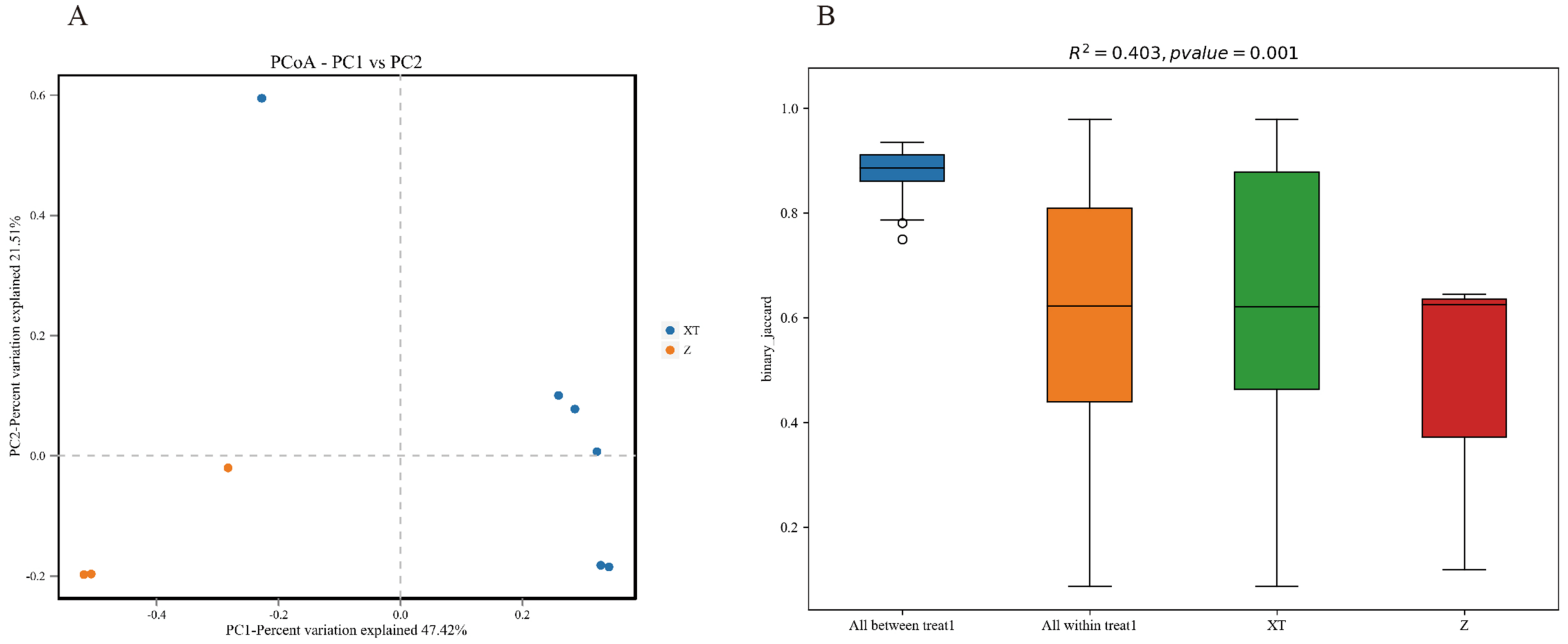
Beta diversity statistical chart. **(A)** Principal coordinate analysis (PCoA) plot of the two species. PC1 and PC2 represent the first and second principal coordinates, respectively. PC1 typically accounts for the highest amount of variance in the dataset, while PC2 captures the second highest variance. Together, they help visualize the relationships and differences among the samples in a reduced-dimensional space. **(B)** PERMANOVA analysis plot.

We compared the microbial diversity across all groups and plotted a principal coordinate analysis (PCoA) graph. The analysis indicates that the Z samples are all located in the third quadrant and are far from the XT samples, suggesting an obvious difference in microbial community composition between the Z and XT samples. PC1 and PC2 explain 47.42 and 21.5% of the variance, respectively, indicating that these two principal components capture a significant portion of the total variability in the data. Specifically, PC1 captured the most variance, reflecting the primary factors driving differences among samples, while PC2 captured additional variability, helping to reveal underlying patterns or groupings in the data (Figure 5A).

PERMANOVA (Adonis), also known as Permutation Multivariate Analysis of Variance, and ANOSIM (Analysis of Similarities), are statistical methods used to analyze the similarity between groups in multidimensional data. PERMANOVA or ANOSIM can test whether there are significant differences in beta diversity between samples from different groups. As shown in Figure 5B, R^2^ represents the proportion of variance explained by grouping, i.e., the ratio of group variance to total variance. A higher R^2^ indicates that grouping explains a larger portion of the variance, and the differences between groups are greater. A *p*-value < 0.05 indicates a high level of confidence in the test results of this study.

### 3.6 Functional gene prediction analysis

**Figure 6:**
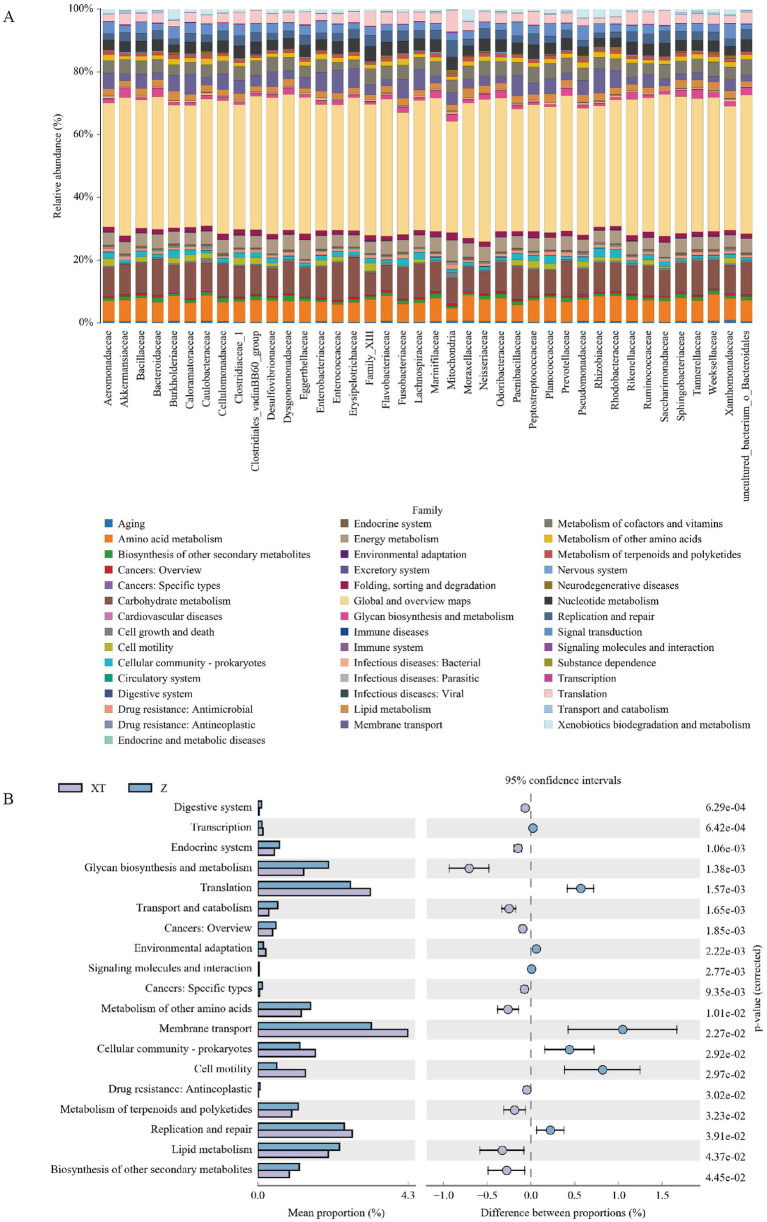
Differences and variations in functional genes of microbial communities in metabolic pathways. **(A)** Bar chart of KEGG metabolic pathways at the family level. **(B)** Analysis of differences in KEGG metabolic pathways between groups, where the left panel shows the abundance ratio of different functions in the two sample groups, the middle panel displays the difference in abundance ratios within a 95% confidence interval, and the rightmost values represent *p*-values.

We also analyzed the composition and differential analysis of Kyoto Encyclopedia of Genes and Genomes (KEGG) metabolic pathways, observing the differences and variations in the functional genes of microbial communities among samples from different groups involved in metabolic pathways. From Figure 6A, we can predict that the metabolic pathways of all families are similar, primarily related to Global and overview maps, Carbohydrate metabolism, Amino acid metabolism, Membrane transport, Energy metabolism, Metabolism of cofactors and vitamins, Signal transduction, Nucleotide metabolism, Cell motility, Lipid metabolism, Translation, Cellular community - prokaryotes, Replication and repair, Metabolism of other amino acids, Folding, sorting and degradation, Glycan biosynthesis and metabolism, Xenobiotics biodegradation and metabolism, and Drug resistance: Antimicrobial.

Figure 6B reveals the differential analysis of KEGG metabolic pathways between the XT and Z groups; compared to the Z group, the XT group showed higher abundances in the pathways of Transcription, Translation, Environmental adaptation, Membrane transport, Cellular community - prokaryotes, Cell motility, and Replication and repair. The differences and variations in the functional genes of microbial communities among samples from different groups in metabolic pathways represent an effective means of studying the metabolic functional changes that occur as communities adapt to environmental changes.

## 4 Discussion

Reptiles are ancient vertebrates, and understanding their gut microbiota can help assess their ecological health and biodiversity, providing important information about their habitat. At the same time, the gut microbiota plays a crucial role in food digestion and nutrient absorption. In reptiles, the composition and function of the gut microbiota can reveal their dietary adaptations and energy utilization efficiency. In terms of disease prevention and control, research on reptilian gut microbiota can identify microbial communities associated with reptile diseases, thereby aiding in the development of new prevention strategies and promoting wildlife conservation. Additionally, comparing the gut microbiota of different reptiles can help us to understand their evolutionary history and adaptive mechanisms, providing data support for phylogenetic studies. Lastly, certain reptilian gastrointestinal microbes may offer insights into human health, particularly in studying the relationship between microorganisms and diseases. Overall, research on the gut microbiota of reptiles not only provides valuable information for basic scientific research but also has practical significance for ecological conservation and public health (Hu et al., 2024).

Currently, there is limited research on the gut microbiota of reptiles. The results of this study indicate that Firmicutes is the most dominant phylum in the XT samples, accounting for 43.31%. In previous research, researchers used 16S rDNA sequencing technology to explore the effects of Long-Snake moxibustion intervention on the gut microbiota of patients with ankylosing spondylitis (AS). The results illustrated that at the phylum level, the relative abundance of Firmicutes and Proteobacteria decreased in the pre-treatment group after Long-Snake moxibustion treatment, whereas the relative abundance of Bacteroidetes and Actinobacteria increased. Ultimately, the findings suggested that Long-Snake moxibustion treatment can improve the clinical symptoms of patients with AS, potentially by regulating the abundance of gut microbiota, increasing beneficial bacteria, and restoring microbial homeostasis (Yu et al., 2024).

Proteobacteria was identified as the most dominant phylum in the Z samples, accounting for 45.13%. Proteobacteria are widely present in various snake species and are typically associated with aquatic environments. Moreover, a 2015 study found an increased prevalence of Proteobacteria in the Cottonmouth Snake, suggesting that its gut microbiome is similar to that of birds (Colston et al., 2015). In addition, our research showed that the abundance of Proteobacteria in the Z group was higher than that in the XT species, leading us to speculate that this may be a result of the Z snake’s habitat near aquatic environments, where they prefer to eat fish or animals near the water.

In addition to the two dominant phyla mentioned above, Bacteroidetes is also a dominant phylum in both types of snakes, and many studies have shown that Bacteroidetes are widely present in wild snakes. Bacteroidetes are anaerobic bacteria that can decompose polysaccharides and enhance nutrient use. They also contribute to the development of the intestinal mucosa and immune system. Bacteroidetes are commensal organisms found in the colon and cloaca that promote the digestion and utilization of carbohydrates (Colston and Jackson, 2016; Spence et al., 2006). However, research on Bacteroidetes is relatively limited, so further exploration by researchers is needed.

The significant roles of these microbial communities were also identified in a study published by Hu et al. (2024), where the researchers collected samples from four brown-spotted pit vipers (*Protobothrops mucrosquamatus*) and three Dione’s rat snakes (*Elaphe dione*) from Mount Laojun, China. The dominant phyla in the feces of these snakes were identified as Bacteroidetes, Proteobacteria, Firmicutes, and Fusobacteria, while the dominant phyla in the oral cavity were Proteobacteria, Bacteroidetes, Actinobacteria, and Firmicutes. Additionally, a 2023 study compared the gene sequence data of gut microbiota from 91 reptile species and found that Bacteroidota, Proteobacteria (mostly Gamma class), and Firmicutes are the predominant bacterial phyla in most reptiles (Hoffbeck et al., 2023). However, the gut microbiota of reptiles exhibits significant variation even among individuals of the same species, although the microbial community differences are even greater between different species. Among these, Bacteroides was identified as the ‘core’ microbiota in most reptiles, which aligns with our findings and provides new insights into the key drivers of reptile gut microbial communities. These two studies are of significant importance in advancing our understanding of the genetics, evolution, and ecology of organisms.

The ecological bacterial communities (e.g., microbial populations in soil and water) and fecal bacterial communities (excreted by animals or humans) exhibit complex and dynamic interactions, involving multiple aspects such as material cycling, gene transfer, and environmental adaptation. Studies have shown that the application of organic fertilizers like cow manure introduces a significant amount of fecal bacteria (e.g., Firmicutes, Actinobacteria) and antibiotic resistance genes (ARGs) into the soil, markedly altering the diversity and functional gene distribution of soil microbial communities (Shen et al., 2023). Additionally, the fecal microbiota of high-altitude populations of the striped plateau lizard (*Sceloporus grammicus*) exhibit heightened activity in amino acid and vitamin metabolic pathways, potentially releasing metabolic intermediates (e.g., short-chain fatty acids) into the environment through excretion, thereby promoting nitrogen fixation by soil microbes and enhancing plant nutrient absorption (Montoya-Ciriaco et al., 2020). In-depth research into these interaction mechanisms not only helps reveal ecological adaptation strategies in extreme environments but also provides a scientific basis for ecological conservation, pollution control, and animal health management.

At the same time, we observed significant differences in the metabolic pathways between the two snake species. Variations in metabolic pathways often reflect functional differences between species, which may be closely related to adaptation to specific environments, metabolic demands, or ecological roles. In addition, changes in metabolic pathways may result from differences in living environments, dietary structures, and external pressures. Differences in microbial community composition may also be a key factor influencing these variations. Distinct metabolic pathways may indicate evolutionary divergence between species or adaptation to specific ecological niches. Therefore, differences in metabolic pathways not only reveal the adaptive mechanisms of organisms but also provide valuable insights into the relationships among environment, nutrition, and function.

During the Beta Diversity Analysis, we observed that the PCoA plot (Figure 5A) does not show clear clustering, indicating significant differences among individuals. This could be attributed to host-specific factors, such as the genetic background, age, or transient physiological states (e.g., immune status, hormone levels) of the snakes. Additionally, environmental micro-heterogeneity might also play a role, as subtle differences in microhabitats within the same treatment group (e.g., soil pH, variations in dietary composition) can drive divergent microbial responses. In future studies, controlling for diet and conducting host genotyping will help dissect these driving factors. Figure 5B further supports this observation, showing a box plot with an *R*^2^ value of 0.403 and a *p*-value of 0.001, indicating significant variation between treatments. The colors and layout of the figure help visualize the distribution of binary_jaccard indices across different groups, highlighting the complexity of microbial community dynamics influenced by both host and environmental factors.

In summary, metagenomic sequencing revealed distinct gut microbial structures between Red Back Pine Root Snakes (*Oligodon formosanus*, XT) and Chinese Slug-Eating Snakes (*Pareas chinensis*, Z). XT was dominated by Proteobacteria, Bacteroidetes, Firmicutes, Verrucomicrobia, Actinobacteria, and Fusobacteria, while Z additionally harbored Patescibacteria. Significant divergence occurred at family level, with higher species diversity in Z. KEGG analysis highlighted differential enrichment in metabolic pathways (e.g., nutrient metabolism, environmental adaptation), likely driven by habitat-specific pressures. These findings underscore the role of ecological adaptation in shaping gut microbiota, providing insights for snake conservation and zoonotic disease research.

## Data Availability

The datasets presented in this study can be found in online repositories. The name of the repository/repositories and accession number(s) can be found below: NCBI - PRJNA1238482.
